# Risk factors for severe maternal morbidity in patients with hypertensive disorder of pregnancy: A retrospective study

**DOI:** 10.12669/pjms.41.7.12023

**Published:** 2025-07

**Authors:** Zhuanji Fang, Huale Zhang, Guizhen Xu, Qinjian Zhang, Liping Huang, Jianying Yan

**Affiliations:** 1 Zhuanji Fang Department of Obstetrics and Gynecology, Fujian Maternity and Child Health Hospital, College of Clinical Medicine for Obstetrics & Gynecology and Pediatrics, Fujian Medical University, 18 Daoshan Road, Fuzhou, Fujian Province 350001, P.R. China; 2 Huale Zhang Department of Obstetrics and Gynecology, Fujian Maternity and Child Health Hospital, College of Clinical Medicine for Obstetrics & Gynecology and Pediatrics, Fujian Medical University, 18 Daoshan Road, Fuzhou, Fujian Province 350001, P.R. China; 3 Guizhen Xu Department of Obstetrics and Gynecology, Fujian Maternity and Child Health Hospital, College of Clinical Medicine for Obstetrics & Gynecology and Pediatrics, Fujian Medical University, 18 Daoshan Road, Fuzhou, Fujian Province 350001, P.R. China; 4 Qinjian Zhang Department of Obstetrics and Gynecology, Fujian Maternity and Child Health Hospital, College of Clinical Medicine for Obstetrics & Gynecology and Pediatrics, Fujian Medical University, 18 Daoshan Road, Fuzhou, Fujian Province 350001, P.R. China; 5 Liping Huang Department of Obstetrics and Gynecology, Fujian Maternity and Child Health Hospital, College of Clinical Medicine for Obstetrics & Gynecology and Pediatrics, Fujian Medical University, 18 Daoshan Road, Fuzhou, Fujian Province 350001, P.R. China; 6 Jianying Yanm Department of Obstetrics and Gynecology, Fujian Maternity and Child Health Hospital, College of Clinical Medicine for Obstetrics & Gynecology and Pediatrics, Fujian Medical University, 18 Daoshan Road, Fuzhou, Fujian Province 350001, P.R. China

**Keywords:** Hypertensive disorder of pregnancy, Iron supplementation, Preeclampsia, Risk factors, Severe maternal morbidity

## Abstract

**Objective:**

To identify significant risk factors associated with severe maternal morbidity(SMM) in patients with hypertensive disorder of pregnancy (HDP).

**Methods:**

This retrospective study analyzed clinical data from patients with HDP who delivered at Fujian Maternity and Children Health Hospital between January 2013 and March 2022. Univariate logistic regression analysis was performed to identify risk factors for developing SMM. Significant risk factors (P < 0.05) were considered for inclusion in multivariate logistic regression using stepwise regression with forward and backward selection.

**Results:**

Of 3133 HDP patients included in the study, 365 met the diagnostic criteria of SMM and were included in the SMM group, while 2768 patients comprised the control group. The SMM group had a significantly higher incidence of gestational hypertension diagnosed at ≤ 34 weeks of gestation compared to the control group (30.14% vs. 12.64%, p<0.0001). Patients in the SMM group had a higher incidence of previous history of preeclampsia compared to the control group (1.64% vs. 0.25%, p=0.0001). Logistic regression analysis identified parity (OR, 1.37; CI, 1.05-1.78; p=0.0205), gestational age of diagnosis (OR, 2.22; CI, 1.68-2.92; p<0.0001), iron supplementation (OR, 2.31; CI, 1.83-2.93; p<0.0001), and preeclampsia (OR, 3.10; CI, 2.42-3.98; p<0.0001) as significant risk factors for SMM. Stepwise regression analysis confirmed that parity (OR, 1.43; CI, 1.17-1.73; p=0.0004), gestational age of diagnosis (OR, 2.32; CI, 1.77-3.05; p<0.0001), iron supplementation (OR, 2.30; CI, 1.82-2.90; p<0.0001), and preeclampsia (OR, 3.34; CI, 2.63-4.24; p<0.0001) remained significantly associated with SMM.

**Conclusion:**

Gestational age of diagnosis, iron supplementation, and history of preeclampsia were identified as risk factors of SMM in patients with HDP. Our results can help identify high-risk patients for early recognition and management of SMM.

## INTRODUCTION

Hypertensive disorders of pregnancy (HDP), including preeclampsia, are associated with severe maternal complications, such as heart attack and stroke, and high mortality.^1^–^3^ Preeclampsia typically occurs after the 20th week of pregnancy. It affects approximately 2-8% of pregnancies worldwide and is considered a leading cause of severe maternal morbidity (SMM) such as preterm delivery, cesarean section, maternal hemorrhage, acute renal failure, and liver dysfunction, especially in low- and middle-income countries where access to obstetric care is limited.^4^,^5^ Therefore, early detection and management of preeclampsia, including prompt delivery in severe cases, are critical in reducing the risks of SMM and mortality.^6^,^7^ Studies have shown that interventions such as timely referral and management of high-risk pregnancies, implementation of evidence-based obstetric care, and improvement of health systems can significantly reduce the risk of SMM.^8^,^9^ This study aimed to identify independent risk factors associated with SMM in patients with HDP.

## METHODS

This retrospective study collected and analyzed clinical data of patients with HDP who were delivered in Fujian Maternity and Children Health Hospital between January 1, 2013 and March 31, 2022 to identify the risk factors associated with SMM.

### Ethical statement:

The study was approved by the ethics committee of Fujian Maternity and Child Health Hospital (Ethical Approval Number: 2020YJ183), Date: September 15, 2020. We confirm that all methods were performed per the ethical standards in the Declaration of Helsinki and its later amendments or comparable ethical standards.

### Study population:

All patients with clinically diagnosed HDP were included. The information on diagnosed chronic hypertension, gestational hypertension, preeclampsia, and eclampsia was extracted from the electronic medical records. During the initial screening, 3133 cases were identified. After excluding patients with severe medical and surgical conditions, patients meeting the criteria for SMM at the time of diagnosis of HDP, and patients with unclear pregnancy outcomes, a total of 3111 cases, were included in the final analysis. The enrolled patients were divided into the SMM group and the control group.

Since there is no global consensus on diagnosing SMM yet, we referred to the list of diagnoses and complications constituting SMM, issued in 2016 by the ACOG and the Society for Maternal Fetal Medicine (SMFM). The diagnostic criteria of SMM were as follows:

- Emergency/unplanned peripartum hysterectomy,
- Obstetric hemorrhage with ≥4 units of red blood cells (or two units of red blood cells and two units of fresh frozen plasma) transfused,
- Preeclampsia with difficult-to-control severe hypertension (>160 systolic blood pressure or >110 diastolic blood pressure) that requires multiple intravenous doses,
- Renal and hepatic insufficiency, sepsis, etc.

### Exclusion Criteria:

All To ensure homogeneity and accuracy in the study population, we applied the following exclusion criteria:

- Pregnant women have serious medical and surgical conditions before they are diagnosed with hypertensive disorders during pregnancy, such as:
- Pre-gestational diabetes mellitus with end-organ damage, heart disease leading to abnormal cardiac function, chronic renal failure, autoimmune diseases.
- Pass-through excluded patients who already met the diagnostic criteria for severe maternal morbidity (SMM) at the time of initial diagnosis of hypertensive disorders of pregnancy.
- Patients with incomplete or unclear pregnancy outcomes.

### Study objective:

The primary objective of this study was to identify significant risk factors associated with SMM in patients with preeclampsia

### Data collection:

Data were collected at the time of delivery, focusing on the gestational age at diagnosis, which ranged from 20 weeks to 41 weeks.

Collected data included baseline information such as age, body mass index (BMI), blood pressure and pulse data, information on para and gravida, and weight gain during pregnancy, information on signs and symptoms such as swelling, dizziness, headache, blurred visions, epigastric discomfort, and chest discomfort, pregnancy complications such as preeclampsia, assisted reproduction, gestational diabetes mellitus, polyhydramnios, stillbirth, and small for gestational age, medication during pregnancy such as iron supplementation, calcium supplementation, vitamins, and heparin, and laboratory investigations such as hemoglobin, platelets, neutrophils, total cholesterol, high-density lipoprotein (HDL), low-density lipoprotein (LDL), triglycerides, albumin, alanine aminotransferase (ALT), aspartate aminotransferase (AST), direct bilirubin (Dbil), indirect bilirubin (Ibil), creatinine, CO2 combining power (CO2CP), fibrinogen, calcium, magnesium, and lactate dehydrogenase (LDH).

### Statistical analysis:

Patients’ baseline characteristics and operation information were described as proportions for categorical variables and as means, standard deviations, medians, interquartile ranges, and ranges for continuous variables. The significance of differences was assessed using the χ2 test (categorical variable), student’s t-test, and Mann-Whitney U test (continuous variable) depending on the data distributions and variances. Statistical significance was set at *P* < 0.05. Univariate logistic regression analysis was conducted to calculate dependent odds ratios (ORs) and determine the risk factors for developing SMM. Risk factors were considered for inclusion in the multivariate logistic and stepwise regression according to clinical experience or significance (*P* <0.05) in the univariate analysis. Stepwise regressions were conducted with forward and backward selection. All analyses were conducted using the SAS System version 9.4 (SAS Institute, Cary NC).

## RESULTS

### Baseline information on the patients:

**Fig.1:**
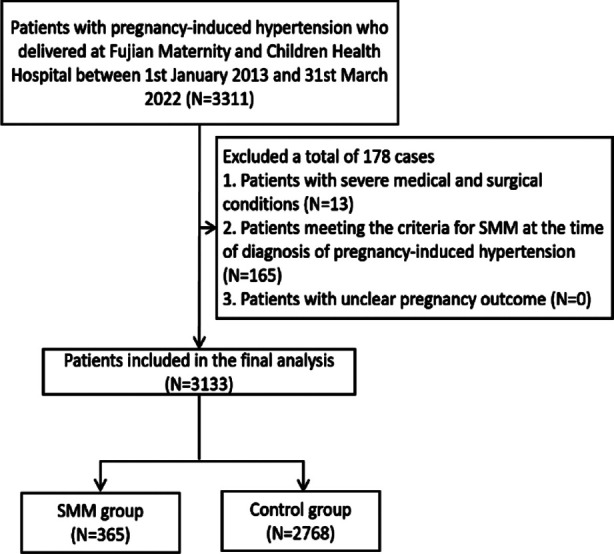
Case-screening flowchart.

A total of 3133 patients were included in the final analysis. Of them, 365 were in the SMM group, and 2768 patients were in the control group (Fig.1).

As summarized in Table-I, most patients were between 20 and 35 years old (79.18% in the SMM group and 86.05% in the control group) (p=0.0011) and had a pre-pregnancy BMI of 18.5-24 (62.69%). In the first trimester, the median systolic blood pressure (SBP) and diastolic blood pressure (DBP) were 121 and 75, respectively. Most patients in both groups gained weight during pregnancy, with a median overall weight gain of 14.4kg (26.2%). In the SMM group, 30.14% of patients were diagnosed with gestational hypertension at ≤ 34 weeks of gestation compared to 12.64% of cases in the control group (*p*<0.0001). Patients in the SMM group had a higher incidence of previous preeclampsia than the control group (1.64% vs. 0.25%, *p*=0.0001).

**Table-I:** Baseline information on the enrolled cases.

Variable	SMM (N=365)	Control (N=2768)	P-value
***Age (years)***			0.0011
<20 years	2 (0.55%)	21 (0.76%)	
20-35 years	289 (79.18%)	2382 (86.05%)	
>35 years	74 (20.27%)	365 (13.19%)	
Pre-pregnancy BMI			0.2127
<18.5	43 (12.01%)	348 (12.91%)	
[18.5-24)	224 (62.57%)	1690 (62.71%)	
[24-28)	59 (16.48%)	491 (18.22%)	
≥28	32 (8.94%)	166 (6.16%)	
SBP (first trimester) (mmHg)	121.51 ± 11.21	121.05 ± 10.48	0.3409
DBP (first trimester) (mmHg)	74.17 ± 8.39	74.23 ± 8.60	0.7811
Gravidity			0.0006
<3	248 (68.70%)	2112 (76.94%)	
≥3	113 (31.30%)	633 (23.06%)	
Parity			0.0002
0	209 (57.89%)	1883 (68.60%)	
1	134 (37.12%)	773 (28.16%)	
>1	18 (4.99%)	89 (3.24%)	

### Clinical features and pregnancy complications:

As shown in Table-II, the SMM group had a higher proportion of patients presenting symptoms than the control group (35.89% vs. 23.63%, *p*<0.0001). Swelling (18.38%) and chest discomfort (4.21%) were the most common overall symptoms. Patients in the SMM group had a significantly higher incidence of swelling (27.78% vs. 17.16%, *p*<0.0001), dizziness (4.11% vs. 2.31%, *p*=0.04), headache (3.56% vs. 1.95%, *p*=0.046), and blurred vision (5.21% vs. 2.315, *p*=0.001).

**Table-II:** Symptoms presentation in the enrolled cases.

Symptom	SMM (N=365)	Control (N=2768)	P-value
Symptom presentation			<0.0001
Yes	131 (35.89%)	654 (23.63%)	
No	234 (64.11%)	2114 (76.37%)	
Swelling			<0.0001
Yes	101 (27.67%)	475 (17.16%)	
No	264 (72.33%)	2293 (82.84%)	
Dizziness			0.04
Yes	15 (4.11%)	64 (2.31%)	
No	350 (95.89%)	2704 (97.69%)	
Headache			0.046
Yes	13 (3.56%)	54 (1.95%)	
No	352 (96.44%)	2714 (98.05%)	
Blurred vision			0.001
Yes	19 (5.21%)	64 (2.31%)	
No	346 (94.79%)	2704 (97.69%)	

As shown in Table-II, preeclampsia (35.97%) and gestational diabetes mellitus (21.80%) were the most common complications overall. The SMM group had a significantly higher incidence of preeclampsia (64.38% vs. 32.23%, *p*<0.0001), stillbirth (5.21% vs. 2.38%, *p*=0.0018), and small for gestational age (11.78% vs. 5.49%, *p*<0.0001).

### Medications during pregnancy:

Most patients had used medications during pregnancy (70.76% vs. 29.24%). As shown in Table-III, vitamins (54.36%), iron supplementation (34.60%), and calcium supplementation (20.65%) were the most used medications overall. Patients in the SMM group had a significantly higher rate of medication use compared to the control group (84.11% vs. 69%, *p*<0.001). Compared to the control group, patients in the SMM group reported higher use of iron supplementation (51.51% vs. 32.37%, *p*<0.001), calcium supplementation (34.25% vs. 18.86%, *p*<0.0001), vitamins (61.37% vs. 53.43%, *p*=0.0042), and low molecular weight heparin (14.52% vs. 2.31%, *p*<0.0001).

**Table-III:** Medications use during pregnancy.

Medication	SMM (N=365)	Control (N=2768)	P-value
Medication during pregnancy			<0.0001
Yes	307 (84.11%)	1910 (69.00%)	
No	58 (15.89%)	858 (31.00%)	
Iron supplementation			<0.0001
Yes	188 (51.51%)	896 (32.37%)	
No	177 (48.49%)	1872 (67.63%)	
Calcium supplementation			<0.0001
Yes	125 (34.25%)	522 (18.86%)	
No	240 (65.75%)	2246 (81.14%)	
Low-molecular-weight-heparin			<0.0001
Yes	53 (14.52%)	64 (2.31%)	
No	312 (85.48%)	2704 (97.69%)	

### Laboratory parameters:

**Supplementary Fig.1:**
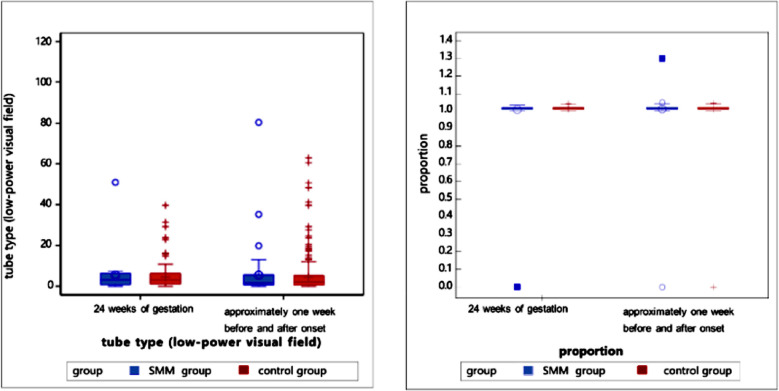
Urine routine tests of pregnant women in the SMM and non-SMM groups at 24 weeks of gestation and one week before or after diagnosis (delivery). Urinary tests are shown on the left, while specific gravity is depicted on the right.

**Supplementary Fig.2:**
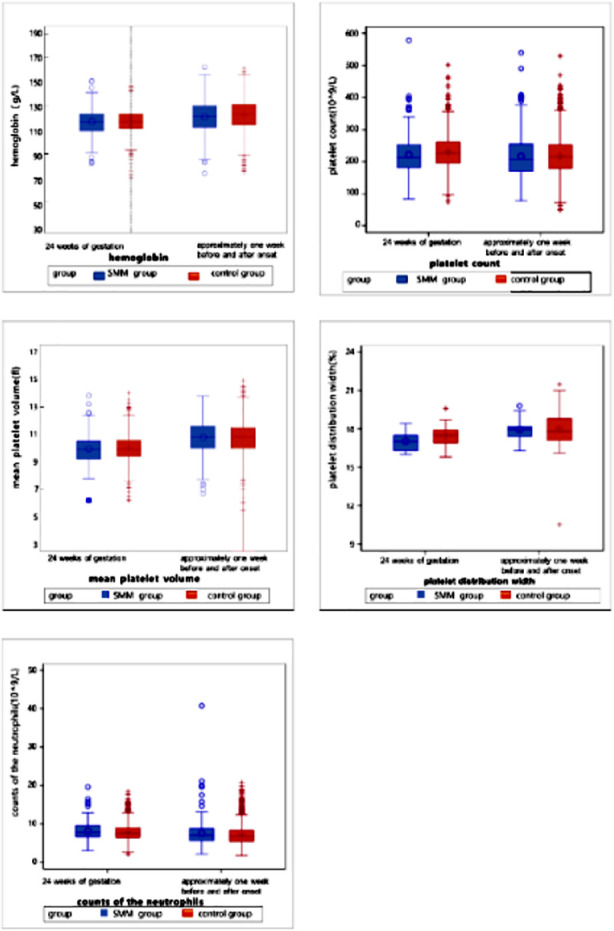
Blood routine tests at 24 weeks of gestation & one week before or after the diagnosis (delivery).

**Supplementary Fig.3:**
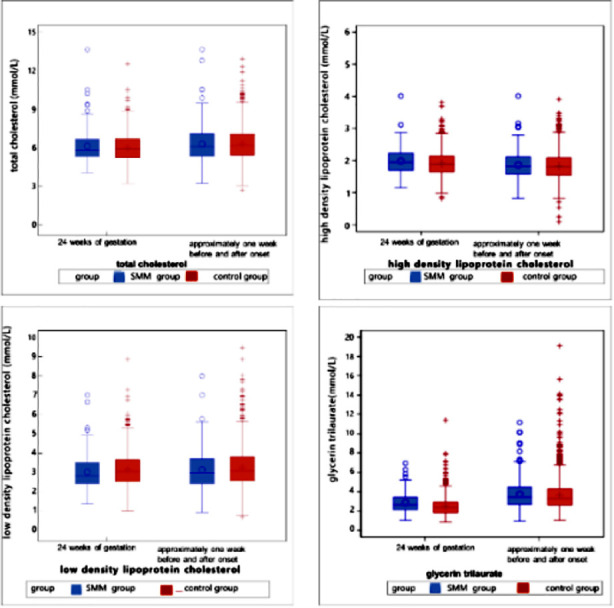
Lipid profiles including total cholesterol, high-density lipoprotein, low-density lipoprotein, and triglycerides at 24 weeks of gestation and one week before or after the diagnosis (delivery).

**Supplementary Fig.4:**
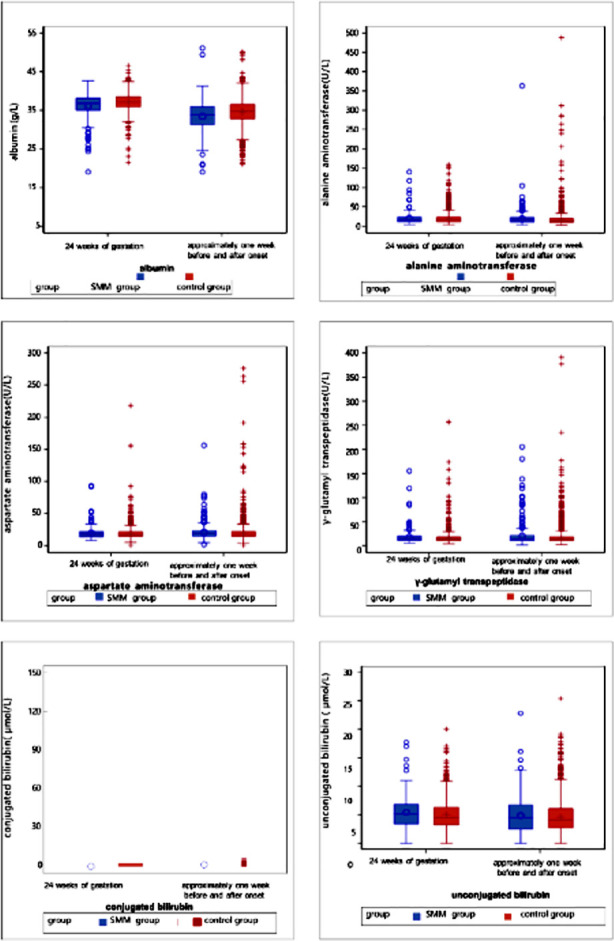
Liver function tests including ALT, GGT, conjugated bilirubin, and unconjugated bilirubin at 24 weeks of gestation and one week before or after the diagnosis (delivery).

**Supplementary Fig.5:**
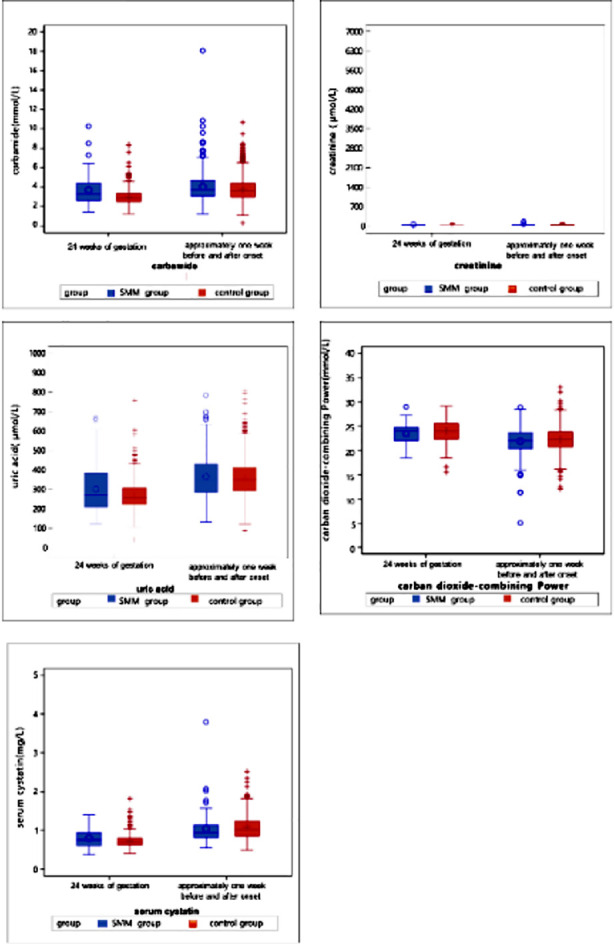
Renal function tests including blood urea nitrogen, creatinine, uric acid, carbon dioxide binding capacity, and serum cystatin C at 24 weeks of gestation and one week before or after the diagnosis (delivery).

**Supplementary Fig.6:**
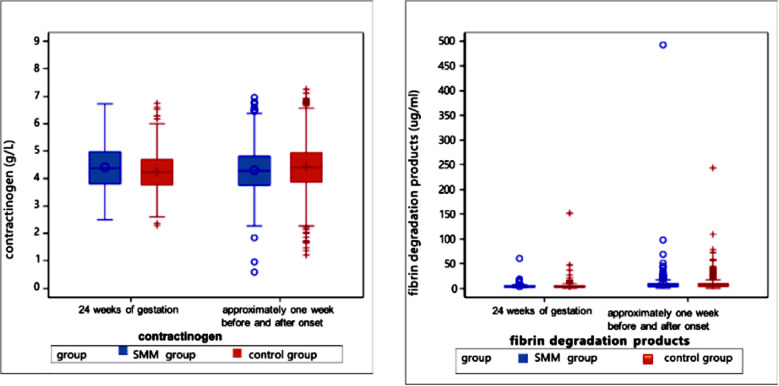
Coagulation function tests including fibrinogen and fibrinogen degradation products at 24 weeks of gestation and one week before or after the diagnosis (delivery).

**Supplementary Fig.7:**
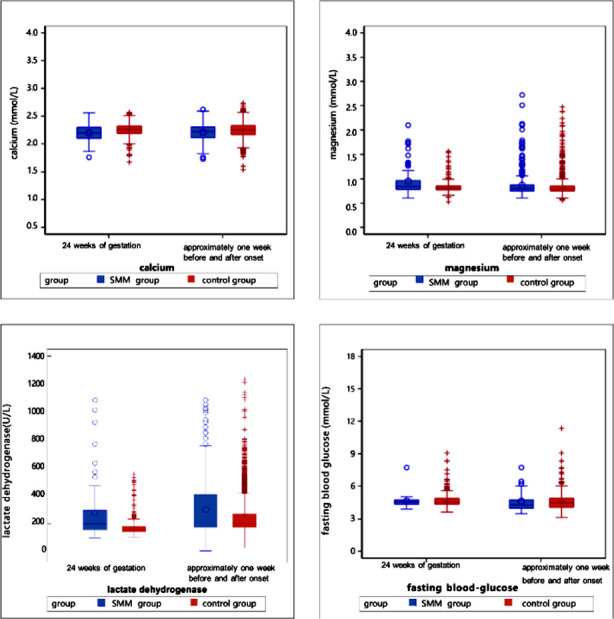
Electrolytes and blood glucose levels at 24 weeks of gestation and one week before or after the diagnosis (delivery). These include serum calcium, serum magnesium, lactate dehydrogenase, and fasting blood glucose.

Laboratory parameters of patients were analyzed one week before and one week after the HDP diagnosis (Table-IV). Compared to the control group, the SMM group had significantly higher levels of neutrophils (7.07 vs. 6.71, *p*=0.009), ALT (15 vs. 13.02, *p*<0.0001), AST (19 vs. 17.5, *p*<0.0001), urea (3.75 vs. 3.61, *p*=0.004), creatinine (53 vs. 49.7, *p*<0.0001), and LDH (207.3 vs. 202.15, *p*=0.028) and significantly lower levels of albumin (33.8 vs. 34.7, *p*<0.0001), cystatin C (0.96 vs. 1.03, *p*=0.038), fibrinogen (4.28 vs. 4.41, *p*=0.004), calcium (2.22 vs. 2.25, *p*<.0001), and FDP (5.73 vs. 6.15, *p*=0.027) (Supplementary Fig.1-7).

**Table-IV:** Multivariable logistic regression analysis.

Variable	Adjusted OR (95% CI)	P-value	Stepwise OR (95% CI)
Parity	1.37 (1.05-1.78)	0.0205	1.43 (1.17-1.73)
Gestational age ≤34w	2.22 (1.68-2.92)	<0.0001	2.32 (1.77-3.05)
Iron supplementation	2.31 (1.83-2.93)	<0.0001	2.30 (1.82-2.90)
Preeclampsia	3.10 (2.42-3.98)	<0.0001	3.34 (2.63-4.24)
LDH	1.64 (1.30-2.07)	<0.0001	1.35 (1.05-1.73)

### Logistic regression analysis:

Logistic regression analysis was used to identify significant risk factors associated with SMM. The univariate logistic regression analysis showed that age, gravidity, parity, gestational age of diagnosis, iron supplementation, preeclampsia, edema, headache, dizziness, or blurred vision, albumin, ALT, creatinine, and lactate dehydrogenase were significantly associated with SMM (Table-IV). These variables were then analyzed by multivariate logistic regression analysis, which revealed that parity (OR, 1.37; CI, 1.05-1.78; *p*=0.0205), gestational age of diagnosis (OR, 2.22; CI, 1.68-2.92; *p*<0.0001), iron supplementation (OR, 2.31; CI, 1.83-2.93; *p*<0.0001), and preeclampsia (OR, 3.10; CI, 2.42-3.98; *p*<0.0001) were the significant risk factors of SMM. A stepwise regression analysis showed that parity (OR, 1.43; CI, 1.17-1.73; *p*=0.0004), gestational age of diagnosis (OR, 2.32; CI, 1.77-3.05; *p*<0.0001), iron supplementation (OR, 2.30; CI, 1.82-2.90; *p*<0.0001), and preeclampsia (OR, 3.34; CI, 2.63-4.24; *p*<0.0001) were significantly associated with SMM (Table-IV).

## DISCUSSION

This population based case control study showed that younger gestational age at onset (< 34 weeks), multiparity (parity ≥2), presence of urinary protein (diagnosis of preeclampsia), and iron supplementation were all independent risk factors for the development of SMM in patients with HDP. Recent population-based studies have shown that the annual growth rate of SMM in China in the past decade has been about 0.3%.^10^,^11^ Due to the significant decrease in maternal mortality, SMM has replaced maternal mortality as one of the leading indicators of obstetric quality management. Since the incidence rate of SMM has increased substantially over the last decade, early diagnosis of potential SMM in pregnant women is critical, and there is an urgent need to identify risk factors of SMM to develop early interventions.

Our study found that the incidence of SMM in patients with HDP was 11.57%. These results are slightly higher than those reported by the extensive study conducted in the United States (US) (8.71%).^12^ This difference may be due to the increased proportion of high-risk pregnant women in tertiary care hospitals.

We found that pregnant women with gestational hypertension at onset < 34 weeks were more likely to develop SMM, highlighting the need for differentiated management of different subtypes. Our observation is similar to the results of previous studies.^13^,^14^ Li B et al.^14^ investigated the relationship between gestational age at the onset of preeclampsia and clinical characteristics and adverse pregnancy outcomes in a retrospective cohort study based on the Chinese population and reported that pregnant women with a younger gestational age at the onset had a significantly higher incidence of kidney damage, higher blood pressure, and adverse maternal and infant outcomes. This result may be due to the heterogeneity in the pathogenesis of different subtypes of preeclampsia. Therefore, the expert consensus categorizes early-onset preeclampsia (delivery < 34 weeks) as severe. von Dadelszen P et al.^15^ suggested that patients with preeclampsia should be divided into two categories: early-onset or late-onset because preeclampsia depends on gestational age (< 34 weeks or ≥34 weeks) at which symptoms appear. According to the “classic” two-stage theory of preeclampsia,^16^ early onset preeclampsia leads to placental malperfusion due to impaired placental vascular development, which is more likely to result in end-organ involvement and SMM. Since the exact pathogenesis and progression of the first stage of “classic” preeclampsia are still unknown, obstetric management is usually limited to close observation and monitoring of blood pressure, laboratory measures, and fetal health, and, if necessary, promoting lung maturation and labor induction to prevent the most severe complications.

We also observed a higher risk of SMM among women who took iron supplementation during pregnancy. Interestingly, previous studies have also found a clear correlation between high iron storage status and the risk of cardiovascular diseases such as hypertension in non-pregnant people.^17^-^19^ Recent meta-analyses^20^,^21^ reported that high serum ferritin levels are associated with an increased risk of HDP, specifically gestational hypertension and preeclampsia. It may be related to the risk of HDP due to the formation of reactive forms of oxygen free radicals and changes in cell function through iron catalysis, namely ferroptosis.^22^ We may speculate that excessive iron supplementation during pregnancy may lead to increased postprandial non-transferrin-bound iron levels and, subsequently, manifestations of placental ferroptosis, such as oxidative stress, lipid peroxidation, and DNA damage. Alternatively, imbalances in placental iron homeostasis during pregnancy may activate ferroptosis. Excess iron accumulation has been found to inhibit maternal hepcidin production or activity, leading to continued absorption of dietary iron, increasing blood viscosity, clogging blood vessels, and reducing placental perfusion.^23^ Zhu et al.^24^ used a preeclampsia rat model to evaluate the effects of ferritin concentration and ferroptosis levels at different stages of pregnancy on placental function. They showed that increasing ferritin levels in the third trimester of pregnancy accelerates ferroptosis and aggravates symptoms of preeclampsia. Furthermore, the effects on iron metabolism may be even more complex, as present correlations cannot account for all the findings. Some studies have also found that maternal iron status in early pregnancy is not associated with gestational hemodynamic adaptation or risk of HDP.^25^ Clinically, careful evaluation of iron supplementation status and iron supplementation practices during pregnancy may be required to mitigate potential risks. However, there are some studies that have the opposite opinion.^26^ In conclusion, while serum ferritin levels are closely related to HDP, further studies are required to elucidate the mechanism of its direct relationship.

Our study shows that multiparity (≥2) increases the risk of SMM. Previous studies have shown that parity has a significant protective effect in terms of adverse neonatal outcomes,^26^-^31^ one which may be related to the faster delivery process. However, there is limited data on maternal disease progression. This study found that parity ≥2 increased the risk of SMM, which may be related to the older age of multipara women and the higher incidence of HDP. It is well known that increasing maternal age has a clear correlation with various adverse pregnancy outcomes, such as miscarriage, preeclampsia, SGA, GDM, and cesarean section.^32^

We found an association between proteinuria and the severity of hypertensive disorders in pregnancy. Our results are similar to the results of previous studies.^33^,^34^ Positive proteinuria may be due to renal injury leading to hypoproteinemia, which is associated with poor prognosis. However, some studies have suggested that hypoproteinemia may appear before clinical manifestations of preeclampsia,^35^,^36^ so the mechanism of positive urinary protein leading to poor prognosis of HDP may need further research to clarify. Future prospective studies are warranted to elucidate these pathways and to evaluate whether early interventions targeting proteinuria can improve maternal outcomes.

This study found that additional high-risk factors may affect the development of SMM, including previous history of preeclampsia and obesity, and their effects may interact with each other. For example, pregnant women with a previous history of preeclampsia have an increased risk of recurrent preeclampsia and preterm birth.^37^ In contrast, women with HDP who deliver prematurely are more likely to develop SMM. In addition, vascular endothelial growth factor-related mononucleotides may be involved in vascular remodeling in pregnant women with a history of preeclampsia, leading to an increased likelihood of adverse cardiovascular events.^38^

### Limitations:

Firstly, as a retrospective, single-center study, there is a risk that incomplete medical records and recall of medical history may lead to selection bias. Additionally, the lack of randomization in either group could introduce imbalance and bias in baseline information. Furthermore, the data included periods before the establishment of standardized protocols for preeclampsia prevention, which may have influenced the observed rates of aspirin use. While the advantage of our study lies in ensuring the consistency of diagnosis and treatment standards, the generalizability of our findings needs to be further confirmed.

## CONCLUSION

This study shows that gestational age of diagnosis, a history of iron supplementation, and preeclampsia are all risk factors for SMM in patients with HDP. Our results can help identify patients who are at high risk for SMM to ensure early recognition, timely management, and better outcomes in this group of women.

## References

[1] Garovic VD, Dechend R, Easterling T, Karumanchi SA, McMurtry Baird S, Magee LA (2022). Hypertension in Pregnancy:Diagnosis, Blood Pressure Goals, and Pharmacotherapy:A Scientific Statement From the American Heart Association. Hypertension.

[2] Petersen EE, Davis NL, Goodman D, Cox S, Mayes N, Johnston E (2019). Vital Signs:Pregnancy-Related Deaths, United States, 2011-2015, and Strategies for Prevention, 13 States, 2013-2017. Morb Mortal Wkly Rep.

[3] Ananth CV, Vintzileos AM (2006). Maternal-fetal conditions necessitating a medical intervention resulting in preterm birth. Am J Obstet Gynecol.

[4] Duley L (2009). The global impact of pre-eclampsia and eclampsia. Semin Perinatol.

[5] Brown MA, Magee LA, Kenny LC, Karumanchi SA, McCarthy FP, Saito S (2018). The hypertensive disorders of pregnancy:ISSHP classification, diagnosis &management recommendations for international practice. Pregnancy Hypertens.

[6] Say L, Chou D, Gemmill A, Tunçalp O, Moller AB, Daniels J (2014). Global causes of maternal death:a WHO systematic analysis. Lancet Glob Health.

[7] Souza JP, Gulmezoglu AM, Vogel J, Carroli G, Lumbiganon P, Qureshi Z (2013). Moving beyond essential interventions for reduction of maternal mortality (the WHO Multicountry Survey on Maternal and Newborn Health):a cross-sectional study. Lancet.

[8] Liu S, Liston RM, Joseph KS, Heaman M, Sauve R, Kramer MS (2007). Maternal mortality and severe morbidity associated with low-risk planned cesarean delivery versus planned vaginal delivery at term. CMAJ.

[9] Say L, Souza JP, Pattinson RC (2009). WHO working group on Maternal Mortality and Morbidity classifications. Maternal near miss--towards a standard tool for monitoring quality of maternal health care. Best Pract Res Clin Obstet Gynaecol.

[10] Tan J, Chen M, Li Y, Liu X, Yu C, Sun X (2014). Modeling to Predict Severe Maternal Morbidity Based on 33993 Deliveries of Registered Study in China. Value Health.

[11] Chen N, Pan J (2022). The causal effect of delivery volume on severe maternal morbidity:an instrumental variable analysis in Sichuan, China. BMJ Glob Health.

[12] Hitti J, Sienas L, Walker S, Benedetti TJ, Easterling T (2018). Contribution of hypertension to severe maternal morbidity. Am J Obstet Gynecol.

[13] An H, Jin M, Li Z, Zhang L, Li H, Zhang Y (2022). Impact of gestational hypertension and pre-eclampsia on preterm birth in China:a large prospective cohort study. BMJ Open.

[14] Li B, Yang H (2022). Comparison of clinical features and pregnancy outcomes in early- and late-onset preeclampsia with HELLP syndrome:a 10-year retrospective study from a tertiary hospital and referral center in China. BMC Pregnancy Childbirth.

[15] von Dadelszen P, Steegers EAP, Duvekot JJ, Pijnenborg R (2010). Pre-eclampsia. Lancet.

[16] Magee LA, Nicolaides KH, von Dadelszen P (2022). Preeclampsia. N Engl J Med.

[17] Tzoulaki I, Brown IJ, Chan Q, Van Horn L, Ueshima H, Zhao L (2008). Relation of iron and red meat intake to blood pressure:cross sectional epidemiological study. BMJ.

[18] Zhu Y, Chen G, Bo Y, Liu Y (2019). Markers of iron status, blood pressure and incident hypertension among Chinese adults. Nutr Metab Cardiovasc Dis.

[19] Stranges S, Guallar E (2008). Dietary iron and blood pressure. BMJ.

[20] Song QY, Luo WP, Zhang CX (2015). High serum iron level is associated with an increased risk of hypertensive disorders during pregnancy:a meta-analysis of observational studies. Nutr Res.

[21] Liu JX, Chen D, Li MX, Hua Y (2019). Increased serum iron levels in pregnant women with preeclampsia:a meta-analysis of observational studies. J Obstet Gynaecol.

[22] Zacharski LR, Chow BK, Howes PS, Shamayeva G, Baron JA, Dalman RL (2007). Reduction of iron stores and cardiovascular outcomes in patients with peripheral arterial disease:a randomized controlled trial. JAMA.

[23] Han D, Jiang L, Gu X, Huang S, Pang J, Wu Y (2020). SIRT3 deficiency is resistant to autophagy-dependent ferroptosis by inhibiting the AMPK/mTOR pathway and promoting GPX4 levels. J Cell Physiol.

[24] Zhu X, Jiang R, Ying X, Li Z, Jiang P (2022). The role of ferritin and iron dextran in exacerbating preeclampsia in an L-NAME-treated rat model. Ann Transl Med.

[25] Taeubert MJ, Wiertsema CJ, Vermeulen MJ, Quezada-Pinedo HG, Reiss IK, Muckenthaler MU (2022). Maternal Iron Status in Early Pregnancy and Blood Pressure Throughout Pregnancy, Placental Hemodynamics, and the Risk of Gestational Hypertensive Disorders. J Nutr.

[26] Shaheen G, Sajid S, Jahan S (2020). Evaluation of coagulation factors and serum ferritin in preeclamptic Pakistani women. J Pak Med Assoc.

[27] Naeh A, Hallak M, Gabbay-Benziv R (2021). Parity and Interval from Previous Delivery-Influence on Perinatal Outcome in Advanced Maternal Age Parturients. J Clin Med.

[28] Košir Pogačnik R, Trojner Bregar A, Lučovnik M, Krajec M, Verdenik I, Blickstein I (2020). The effect of interaction between parity, gestational diabetes, and pregravid obesity on the incidence of preeclampsia. J Matern Fetal Neonatal Med.

[29] Ling HZ, Guy GP, Bisquera A, Poon LC, Nicolaides KH, Kametas NA (2019). The effect of parity on longitudinal maternal hemodynamics. Am J Obstet Gynecol.

[30] Li C, Binongo JN, Kancherla V (2019). Effect of Parity on Pregnancy-Associated Hypertension Among Asian American Women in the United States. Matern Child Health J.

[31] Gold RA, Gold KR, Schilling MF, Modilevsky T (2014). Effect of age, parity, and race on the incidence of pregnancy associated hypertension and eclampsia in the United States. Pregnancy Hypertens.

[32] Khalil A, Syngelaki A, Maiz N, Zinevich Y, Nicolaides KH (2013). Maternal age and adverse pregnancy outcome:a cohort study. Ultrasound Obstet Gynecol.

[33] Chen H, Tao F, Fang X, Wang X (2016). Association of hypoproteinemia in preeclampsia with maternal and perinatal outcomes:A retrospective analysis of high-risk women. J Res Med Sci.

[34] Xie YJ, Peng R, Han L, Zhou X, Xiong Z, Zhang Y (2016). Associations of neonatal high birth weight with maternal pre-pregnancy body mass index and gestational weight gain:a case-control study in women from Chongqing, China. BMJ Open.

[35] Takahashi H, Hisano M, Sago H, Murashima A, Yamaguchi K (2014). Hypoproteinemia in the second trimester among patients with preeclampsia prior to the onset of clinical symptoms. Hypertens Pregnancy.

[36] Sheikh S, Haq G, Kazi S (2015). Frequency of preterm delivery in proteinuric verses non proteinuric pregnancy induced hypertension. J Pak Med Assoc.

[37] Hernández-Díaz S, Toh S, Cnattingius S (2009). Risk of pre-eclampsia in first and subsequent pregnancies:prospective cohort study. BMJ.

[38] Lin A, Pehrson M, Sarno G, Fraser A, Rich-Edwards JW, Gon?alves I (2022). Coronary Artery Restenosis in Women by History of Preeclampsia. J Am Heart Assoc.

